# Three-Dimensional Vanadium and Nitrogen Dual-Doped Ti_3_C_2_ Film with Ultra-High Specific Capacitance and High Volumetric Energy Density for Zinc-Ion Hybrid Capacitors

**DOI:** 10.3390/nano14060490

**Published:** 2024-03-08

**Authors:** Xinhui Jin, Siliang Yue, Jiangcheng Zhang, Liang Qian, Xiaohui Guo

**Affiliations:** Key Laboratory of Synthetic and Natural Functional Molecule Chemistry of Ministry of Education, The College of Chemistry and Materials Science, Northwest University, Xi’an 710069, China; jinxinhui@stumail.nwu.edu.cn (X.J.); yuesiliang98@163.com (S.Y.); zjc15433@163.com (J.Z.); 17613922518@163.com (L.Q.)

**Keywords:** Ti_3_C_2_, doping, three-dimensional structure, hybrid supercapacitors, specific capacitance

## Abstract

Zinc-ion hybrid capacitors (ZICs) can achieve high energy and power density, ultralong cycle life, and a wide operating voltage window, and they are widely used in wearable devices, portable electronics devices, and other energy storage fields. The design of advanced ZICs with high specific capacity and energy density remains a challenge. In this work, a novel kind of V, N dual-doped Ti_3_C_2_ film with a three-dimensional (3D) porous structure (3D V-, N-Ti_3_C_2_) based on Zn-ion pre-intercalation can be fabricated via a simple synthetic process. The stable 3D structure and heteroatom doping provide abundant ion transport channels and numerous surface active sites. The prepared 3D V-, N-Ti_3_C_2_ film can deliver unexpectedly high specific capacitance of 855 F g^−1^ (309 mAh g^−1^) and demonstrates 95.26% capacitance retention after 5000 charge/discharge cycles. In addition, the energy storage mechanism of 3D V-, N-Ti_3_C_2_ electrodes is the chemical adsorption of H^+^/Zn^2+^, which is confirmed by ex situ XRD and ex situ XPS. ZIC full cells with a competitive energy density (103 Wh kg^−1^) consist of a 3D V-, N-Ti_3_C_2_ cathode and a zinc foil anode. The impressive results provide a feasible strategy for developing high-performance MXene-based energy storage devices in various energy-related fields.

## 1. Introduction

With the increasing demand for electronic devices and electric vehicles, various energy storage systems (e.g., lithium-ion batteries, supercapacitors, and metal-ion hybrid capacitors) with excellent electrochemical performance play an important role in modern society [1,2,3,4,5,6,7,8,9,10,11,12]. Among these, lithium-ion batteries have achieved extensive commercial application in various fields [13,14,15,16]. Nevertheless, the marketable applications of lithium-ion batteries are still limited by the availability and price of lithium metal and the safety problems of organic electrolytes. As a result, various metal-ion hybrid capacitors are widely favored for their ultralong cycle life, reliable safety, and high energy density [17,18,19,20,21,22,23]. In particular, zinc-ion capacitors (ZICs) are considered as potential energy storage devices because of the high theoretical specific capacity of their zinc anodes (820 mAh g^−1^ and 5854 mAh cm^−3^), their environment-friendly nature, their robust stability, and their high level of safety [24,25,26,27].

Although ZICs exhibit good energy storage features compared to other conventional supercapacitors, some key technology parameters (e.g., low specific capacity and energy density) need to be further improved. The exploration of new cathode materials with high specific capacity and fast ion transport speed is urgently required [28,29,30,31,32]. At present, the cathode materials of ZICs mainly include carbon-based materials (activated carbon, hollow carbon spheres, and porous carbon) [33,34], TiN [35], conductive polymers [36], transition metal compounds [37], etc. However, the low specific capacity of these reported cathode materials severely limits the energy density of ZICs. In an attempt to improve the energy storage performance of carbon-based materials, Yan et al. designed N-doped porous carbon with a capacity of 136.8 mAh g^−1^ [38] for Zn//N-HPC zinc-ion devices with an extraordinary energy density (191 Wh kg^−1^). Lu et al. fabricated ZICs with both ultra-high energy density (107.3 mAh g^−1^) and excellent cycle lifetime (99.7% retention after 20,000 cycles) by utilising N-doped hierarchically porous carbon [39]. 

In recent years, an emerging two-dimensional material, MXene, has been investigated as an extremely promising cathode material for ZICs due to its intercalation pseudocapacitance mechanism, metallic-like conductivity, and variable surface modification [40,41,42,43,44]. The inevitable agglomerate stacking of MXene nanosheets reduces electrochemically active sites, limiting electrolyte ion diffusion, and prolongs the ion transport distance. To date, the aggregation and stacking problems of MXene have been solved by constructing a 3D structure, and the introduction of additional pseudocapacitance by heteroatom doping can improve MXene energy storage performance. For example, Zhang et al. demonstrated a 3D H-MXene hierarchical pore-structured film using H^+^ crosslinking. The specific capacity of the assembled Zn//3D-PHMF capacitor is 105 mAh g^−1^ at 0.2 mA g^−1^ [45]. Mateen et al. presented N-functionalization Ti_3_C_2_T_x_ in situ by means of hydrothermal reaction. The N-Ti_3_C_2_T_x_ exhibited an unrivalled specific capacitance of 583 F g^−1^ at 1 A g^−1^ [46]. N doping improved the surface wettability and electrical conductivity of MXene and greatly enhanced its electrochemical performance in supercapacitors. Gao et al. synthesized V-doped Ti_3_C_2_T_x_ using a hydrothermal method, which presented excellent specific capacitance (365.9 F g^−1^) at 10 mV s^−1^ and excellent stability of 95% (after 5000 cycles) [47]. Vanadium doping did not change the 2D morphology of MXene and resulted in stronger alkali metal-ion–O interactions on the MXene surface, introducing more active sites. Although these efforts have achieved the increased specific capacity of Ti_3_C_2_T_x_, the relatively low specific capacity and low energy density have severely hindered the practical applications of Ti_3_C_2_ in ZICs. 

Larger layer spacing can significantly increase the shuttle depth of electrolyte ions in Ti_3_C_2_, and ion pre-intercalation can expand the interlayer spacing, which is favorable for ion diffusion. Therefore, we present a kind of V, N dual-doped Ti_3_C_2_ film with a 3D porous structure (3D V-, N-Ti_3_C_2_) based on Zn^2+^ pre-intercalation via a three-step synthetic process involving HCl etching, hydrothermal doping, and Zn^2+^-induced gel. The prepared 3D V-, N-Ti_3_C_2_ film as a cathode displays an outstanding specific capacitance of ~855 F g^−1^ (309 mAh g^−1^) at 0.3 A g^−1^ with an excellent cycling stability. Additionally, the assembled Zn//3D V-, N-Ti_3_C_2_ capacitors deliver an ultra-high energy density (103 Wh kg^−1^), which is almost optimal compared with the currently reported ZICs. This work highlights the potential of the MXene-based materials in metal-ion capacitor systems and other energy storage fields.

## 2. Experimental Section

### 2.1. Preparation of Delaminated Ti_3_C_2_T_x_

Delaminated Ti_3_C_2_T_x_ suspensions were produced from Ti_3_AlC_2_ powder using fluoride etching and an ultrasonic exfoliation process. Briefly, 1.6 g of LiF (Aladdin, 99%) was dissolved into 20 mL 9 M HCl and stirred for 5 min. Then, 1 g Ti_3_AlC_2_ powder was slowly added to the above mixed solution, and an oil bath at 60 °C was used for 48 h to remove Al from the Ti_3_AlC_2_ phase. The solution was then brought to room temperature and washed with deionized water using centrifugation until the pH was close to 7. After ultrasonic treatment under Ar for 2 h and centrifugation at 3500 rpm for 1 h, dark green supernatant was collected. The concentration of Ti_3_C_2_ was determined by drying a certain volume of the solution and weighing its mass.

### 2.2. Preparation of 3D V-, N-Ti_3_C_2_ Film

The 3D V-, N-Ti_3_C_2_ film was obtained via a hydrothermal method and freeze-drying process. Firstly, 20 mg Ti_3_C_2_ was dissolved in 10 mL DI water to obtain Ti_3_C_2_ suspension. Then, 2 g urea and 2 mg ammonium metavanadate (NH_4_VO_3_) was added into the Ti_3_C_2_ suspension; it was stirred for 15 min, and then transferred to a 15 mL Teflon-lined autoclave and maintained at 120 °C for 24 h. The solution was centrifuged and rinsed with deionized water several times. The V, N double-doped Ti_3_C_2_ film (V-, N-Ti_3_C_2_) was then obtained by vacuum filtration and vacuum drying. V-doped Ti_3_C_2_ film (V-Ti_3_C_2_) was prepared in the absence of urea according to the above procedure. Subsequently, 20 mg V-, N-Ti_3_C_2_ was dispersed into 20 mL deionized water and 0.6 mL ZnSO_4_ (1 mg mL^−1^) was added; the solution was then stirred for 4 h. The 3D V-, N-Ti_3_C_2_ film was obtained by means of vacuum filtration and freeze-drying. For comparison, 3D Ti_3_C_2_ film was also obtained by replacing V-, N-Ti_3_C_2_ with pure Ti_3_C_2_.

### 2.3. Assembly of 3D V-, N-Ti_3_C_2_//Zn Device

Typical 3D V-, N-Ti_3_C_2_//Zn devices were constructed using sample 3D V-, N-Ti_3_C_2_ film as the cathode, Zn metallic sheet as the anode, and a glass fiber membrane as the separator. Here, 2 M ZnSO_4_ was used as the electrolyte in the experiments.

## 3. Results and Discussion

**Figure 1:**
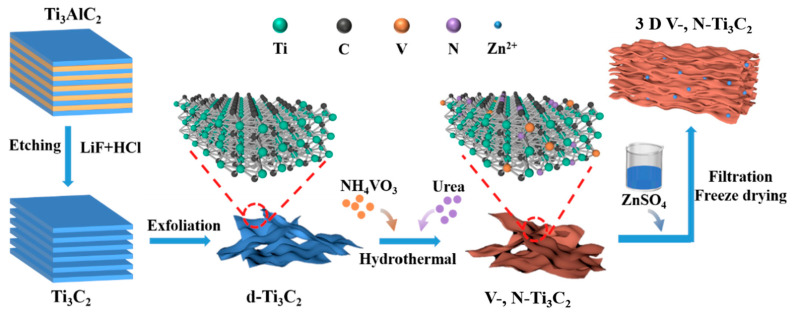
Schematic illustration of the synthesis process of the 3D V-, N-Ti_3_C_2_ film.

The schematic fabrication process of 3D V-, N-Ti_3_C_2_ film is presented in Figure 1. Firstly, few-layered Ti_3_C_2_T_x_ nanosheets were synthesized by etching Al from Ti_3_AlC_2_ in LiF and HCl mixed solution, and then ultrasonic exfoliation was performed, the SEM images of which are shown in Figure S1. Subsequently, the exfoliated Ti_3_C_2_ was then doped with V, N elements via the hydrothermal method in the presence of NH_4_VO_3_ and urea. V-, N-Ti_3_C_2_ was obtained by washing with deionized water. To increase the contact area, we added ZnSO_4_ to V-, N-Ti_3_C_2_ suspension to enable Zn^2+^ ions to destroy the electrostatic repulsive forces between the nanosheets so as to link them together, forming a stable 3D structure. Subsequently, the 3D V-, N-Ti_3_C_2_ film was obtained using vacuum filtration and freeze-drying processes.

**Figure 2:**
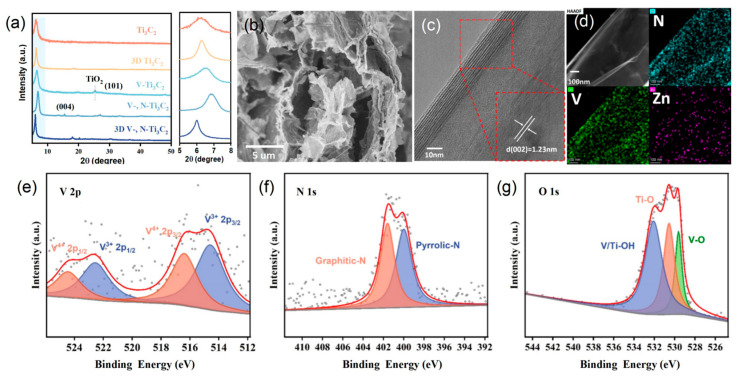
(**a**) XRD patterns of different Ti_3_C_2_-based films. (**b**) Cross-sectional SEM image of the 3D V-, N-Ti_3_C_2_ film. (**c**) HRTEM image of 3D V-, N-Ti_3_C_2_. (**d**) EDX elemental mapping of N, V, and Zn for the 3D V-, N-Ti_3_C_2_. The XPS spectra of (**e**) V 2p, (**f**) N 1s, and (**g**) O 1s for the 3D V-, N-Ti_3_C_2_ film.

The XRD patterns of 3D V-, N-Ti_3_C_2_ and other samples are presented in Figure 2a and Figure S1b. The Ti_3_C_2_ film has an intense (002) peak at around 6°, which confirms that Ti_3_C_2_ was successfully synthesized from Ti_3_AlC_2_. The XRD pattern of 3D V-, N-Ti_3_C_2_ shows that the doping and structural design had minimal effect on Ti_3_C_2_ phase structure. The SEM images (Figures S1–S4 and Figure 2b) showed the morphology of Ti_3_C_2_ film, 3D Ti_3_C_2_ film, V-Ti_3_C_2_ film, V-, N-Ti_3_C_2_ film, and 3D V-, N-Ti_3_C_2_ film. The SEM images of cross-sections of Ti_3_C_2_ film are shown in Figures S2 and S4a,b. V-Ti_3_C_2_ films and V-, N-Ti_3_C_2_ films show that the films are formed by the dense packing of Ti_3_C_2_ nanosheets. The V-Ti_3_C_2_ film and V-, N-Ti_3_C_2_ film are structurally dense and have small amounts of TiO_2_ on the nanosheets’ surface. The dense structure severely limits the exposure of surface active sites and ion diffusion, which in turn affects the electrochemical properties of the films. As for the 3D Ti_3_C_2_ film (the thickness is about 900 nm) and the 3D V-, N-Ti_3_C_2_ film (Figure 2b and Figure S3), stable 3D structures (the thickness is about 45 μm) are constructed by linking zinc ions (Zn^2+^) and by using freeze-drying treatment. The stable 3D structure provides abundant ion channels for membrane electrodes, which dramatically improves their energy storage property. In addition, the nitrogen adsorption–desorption isotherms of V-, N-Ti_3_C_2_, and 3D V-, N-Ti_3_C_2_ samples were of type I and type IV, respectively, as shown in Figure S5, indicating the presence of mesopores in V-, N-Ti_3_C_2_ and both micropores and mesopores in 3D V-, N-Ti_3_C_2_. The surface areas of V-, N-Ti_3_C_2_ and 3D V-, N-Ti_3_C_2_ were 1.23 m^2^ g^–1^ and 45.17 m^2^ g^–1^, respectively. The 3D V-, N-Ti_3_C_2_ film had a larger specific surface area and provided abundant ion adsorption sites and transport channels. Then, TEM was used to observe the morphology and structure of 3D V-, N-Ti_3_C_2_, as shown in Figure S6a. The 3D V-, N-Ti_3_C_2_ nanosheets were intertwined and connected with each other. The HRTEM image (Figure 2c) indicated that the crystal plane spacing of 3D V-, N-Ti_3_C_2_ was 1.23 nm, which was consistent with the (002) plane. The EDS elemental mapping analysis in Figure 2d confirms the coexistence of V, N, and Zn elements in the sample. The uniform distribution of these elements proves that V and N were successfully doped into Ti_3_C_2_.

XPS was employed to investigate the elemental content and chemical state of 3D V-, N-Ti_3_C_2_. The survey of XPS spectra and the high-resolution V 2p spectra of 3D V-, N-Ti_3_C_2_ are given in Figure S7 and Figure 2e, respectively; they demonstrate the successful doping of V into Ti_3_C_2_. The peaks at 516.4 and 524.5 eV correspond to 2p_3/2_ and 2p_1/2_ of V^4+^, respectively [47]. The peaks at a binding energy of 514.6 and 522.5 eV are assigned to 2p_3/2_ and 2p_1/2_ of V^3+^, respectively. The N 1s spectra are shown in Figure 2f, wherein the peaks at 400.0 and 401.6 eV correspond to pyrrolic-N and graphitic-N, respectively, indicating that N was successfully doped into Ti_3_C_2_ [46,48,49]. Furthermore, a high-resolution O 1s spectrum can be simulated to synthesize three peaks; the peaks at 529.6, 530.5, and 532.1 eV correspond to O-V, O-Ti, and HO-V/Ti (Figure 2g), respectively [45]. The peak located at 282.0 eV in the C 1s spectrum (Figure S8b) can be attributed to the C-V, which provides evidence that V is successfully doped into Ti_3_C_2_.

**Figure 3:**
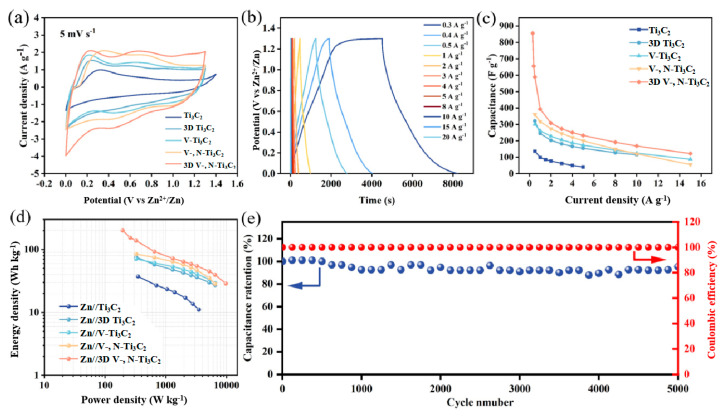
(**a**) CV curves of the Ti_3_C_2_ film, 3D Ti_3_C_2_ film, V-Ti_3_C_2_ film, V-, N-Ti_3_C_2_ film, and 3D V-, N-Ti_3_C_2_ film at 5 mV s^−1^. (**b**) GCD curves of the 3D V-, N-Ti_3_C_2_ film electrode from 0.3 to 20 A g^−1^. (**c**) Specific capacitance of all film electrodes at various current densities. (**d**) Energy and power density profiles for all film electrodes. (**e**) Cycling stability of the 3D V-, N-Ti_3_C_2_ film at 8 A g^−1^.

The Zn^2+^ storage properties of 3D V-, N-Ti_3_C_2_ and other electrode films were then investigated. Figure 3a displays the CV curves of Ti_3_C_2_, 3D Ti_3_C_2_, V-Ti_3_C_2_, V-, N-Ti_3_C_2_, and 3D V-, N-Ti_3_C_2_ at 0–1.3 V when the scanning rate was 5 mV s^−1^. These CV curves have similar shapes, and there are some redox peaks (around 0.2–0.4 V and 0.7–1 V) during charge and discharge, indicating the reversible insertion/removal of Zn^2+^. However, under the same voltage window and scanning rate, the CV areas are in the order Ti_3_C_2_ < 3D Ti_3_C_2_ < V-Ti_3_C_2_ < V-, N-Ti_3_C_2_ < 3D V-, N-Ti_3_C_2_, which indicates that the capacity of 3D-structured MXene is higher than that of MXene film, and the optimal energy storage performance of 3D V-, N-Ti_3_C_2_ is mainly due to the rational creation of the 3D structure that can expand the contact area with the electrolyte ions and then expose more active sites. Additionally, the V-Ti_3_C_2_ and V-, N-Ti_3_C_2_ showed an improved electrochemical performance compared with that of Ti_3_C_2_; this is related to the dual-doping of V and N, which provide additional active sites. Figures S9–S13 show the CV curves of samples in 5~100 mV s^−1^. With the increase in the scanning rate, the CV shape remains unchanged, indicating excellent high-speed and velocity performance. Figure 3b shows the GCD curves of the 3D V-, N-Ti_3_C_2_ at 0.3 to 20 A g^−1^. Then, the GCD curves of Ti_3_C_2_, 3D Ti_3_C_2_, V-Ti_3_C_2_, and V-, N-Ti_3_C_2_ at different current density are shown in Figures S9–S12. The correlation between the gravimetric specific capacitance and current density for all the samples is given in Figure 3c. Similarly to the CV results, the 3D V-, N-Ti_3_C_2_ delivers the highest gravimetric capacitance at 0.3 A g^−1^, up to 855 F g^−1^ (309 mAh g^−1^, Figure S13), and significantly more than the other samples. The gravimetric capacitance of 3D V-, N-Ti_3_C_2_ up to 122 F g^−1^ at 15 A g^−1^ is also better than that of the other electrodes and previously reported Ti_3_C_2_-based electrode materials (Table S1). Results demonstrate that a stable 3D structure can provide a larger active specific surface area and more ion transport channels, while the V, N dual-doping can provide more active sites for the improved capacitance. Remarkably, the 3D V-, N-Ti_3_C_2_ film electrode shows a maximum energy density of 200 Wh kg^−1^ at a power density of 195 W Kg^−1^ (Figure 3d), which is much higher than those of the previously reported zinc-ion capacitors [42,43,45,49]. Furthermore, the cycling stability of the 3D V-, N-Ti_3_C_2_ film electrode was tested at a current density of 8 A g^−1^ (Figure 3e); 95.26% of its initial specific capacitance was retained after 5000 cycles, indicating robust long-term cycle stability. Figure S15 shows linear plots of the resultant charging current density and relevant scan rate for Ti_3_C_2_, 3D Ti_3_C_2_, V-Ti_3_C_2_, V-, N-Ti_3_C_2_, and 3D V-, N-Ti_3_C_2_, where the slopes are the electrochemical double layer capacitance (C_dl_). The 3D V-, N-Ti_3_C_2_ with hierarchical holes had a large ECSA of 7.7 mF/cm^2^, indicating that a large number of electrochemically active sites appeared in the material after V, N co-doping following Zn^2+^ pre-layering.

**Figure 4:**
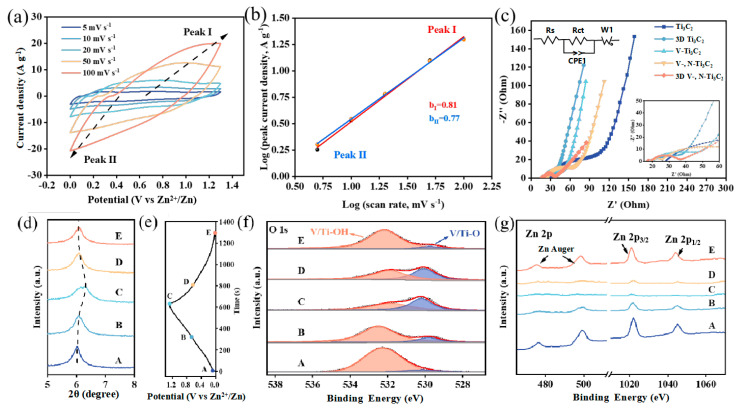
(**a**) CV curves of the 3D V-, N-Ti_3_C_2_ at 5 to 100 mV s^−1^; (**b**) the determination of the b values of the peak I and peak II based on log(i) versus log(v) plots; (**c**) Nyquist plots of 3D V-, N-Ti_3_C_2_ film, Ti_3_C_2_ film, 3D Ti_3_C_2_ film, V-Ti_3_C_2_ film, and V-, N-Ti_3_C_2_ film; (**d**) ex situ XRD patterns of the 3D V-, N-Ti_3_C_2_ cathode; (**e**) GCD profile of 3D V-, N-Ti_3_C_2_; (**f**) ex situ C 1s XPS spectra of the 3D V-, N-Ti_3_C_2_ cathode; and (**g**) ex situ Zn 2p XPS spectra of the 3D V-, N-Ti_3_C_2_ cathode.

To analyse the kinetics of the charge storage of the 3D V-, N-Ti_3_C_2_ electrode, the CV curves of the 3D V-, N-Ti_3_C_2_ at 5–100 mV s^−1^ are presented in Figure 4a. There is a pair of weak redox peaks on the CV curves, and the oxidation peaks (peak I) and reduction peaks (peak II) are gradually shifted towards the positive and negative directions with the increase in the scanning rate. The measured peak current (*i*) and scan rate (*v*) are measured based on the following relationship:

i = av^b^ (1)

where *a* and *b* are variable parameters and *υ* is the scan rate. The *b* value reflects the charge storage mechanism. If the *b* = 0.5, it is a diffusion-controlled process, whereas if the *b* = 1.0, the capacitor dominates the controlled process. The *b* value is the slope of linear fitting of log(*i*) versus log (*v*). As displayed in Figure 4b, the b value of peak Ⅰ is 0.77, and of peak Ⅱ it is 0.81, which suggests a hybrid capacitive and diffusion-controlled charge storage reaction mechanism.

We further analysed the electrochemical kinetic processes with EIS (Figure 4c). The intercepts on the X-axis indicate the electron transfer resistances (R_s_) in the high-frequency region. The R_s_ of Ti_3_C_2_, 3D Ti_3_C_2_, V-Ti_3_C_2_, V-, N-Ti_3_C_2_, and 3D V-, N-Ti_3_C_2_ are measured as 28.2, 20.6, 18.9, 19.3 and 16.5 Ω, respectively. After constructing the 3D structure, the R_s_ of 3D Ti_3_C_2_ and 3D V-, N-Ti_3_C_2_ are decreased to 19.3 and 16.5 Ω, respectively. This indicates that the 3D structure provides more ion transport channels and a larger specific surface area, which effectively reduce solution resistance and electrode resistance. Moreover, V-Ti_3_C_2_ and V-, N-Ti_3_C_2_ show smaller R_s_ values (18.9 and 19.3 Ω) than Ti_3_C_2_ (28.2 Ω), an indication of an enhancement of the conductivity of Ti_3_C_2_ by means of V, N dual-doping. The diameter of the semicircle on the X-axis exhibits charge transfer resistance (R_ct_). The smaller R_ct_ of 3D V-, N-Ti_3_C_2_ (37.1 Ω) indicates that the 3D structure provides more charge transfer channels, while the V/N dual-doping effectively reduces the R_ct_ of Ti_3_C_2_.

To understand the electrochemical reaction mechanism of the 3D V-, N-Ti_3_C_2_ cathode in ZIC devices, we further investigated the structural changes in 3D V-, N-Ti_3_C_2_ during charge–discharge processes by performing ex situ XRD and ex situ XPS. As shown in Figure 4e, based on the GCD curves of Zn//3D V-, N-Ti_3_C_2_ material and taking five representative locations (A, B, C, D, and E) of the cathode, we extracted the surface changes of samples under different charging/discharging conditions. The device was first charged from state A (0 V) to 1.3 V (state C) and then discharged to 0 V (state E) under a constant charging–discharging current. Figure 4d shows the XRD in different states. Firstly, when charging to 0.8 V from 0 V, the peak located at 6.02° indicated that the (002) plane of 3D V-, N-Ti_3_C_2_ experienced a small shift towards a higher angle at 6.07°. Continuing to charge from 0.8 V (state B) to 1.3 V, the (002) peak shifted significantly from 6.07° to 6.29°. This was mainly due to H^+^/Zn^2+^ de-intercalation during the charging process, which caused a reduction in interlayer spacing. Furthermore, the (002) peak shifted from 6.29° to 6.09° with discharge from 1.3 to 0.8 V (state D), then, with discharge from 0.8 V to 0 V (state E), the (002) peak shifted from 6.09° to 6.05°. This was mainly caused by H^+^/Zn^2+^ intercalation during the discharge process [50].

To capture more details of the reaction mechanism, the analysis was performed with ex situ XPS O 1s spectra (Figure 4f). The peaks at 532.6 and 531.6 eV at initial state belong to the V/Ti-OH and V/Ti-O groups, respectively. In the charge steps, the characteristic V/Ti-OH peak intensity at 532.6 eV undergoes a significant decrease from 0 to 0.8 V. As the charge process rises to 1.3 V, there is a slight decrease in the intensity of the characteristic peak of V/Ti-OH. In the discharge process, the intensity of the V/Ti-OH characteristic peak decreases slightly when the discharge voltage ranges from 1.3 V to 0.8 V, then manifests a sharp decrease following a further discharge to 0 V, which is related to the redox reaction of H^+^. To further investigate the contribution of Zn^2+^ in the charge/discharge process, the ex situ XPS Zn 2p spectra are shown in Figure 4g. Herein, the characteristic peak intensity of Zn 2p and the Auger lines of Zn decline in the charge process (from state A to state C) and then gradually increase during the discharge stage (from state C to state E). This phenomenon illustrates the electrochemical reaction of Zn^2+^ with the oxygen terminals on the surface of 3D V-, N-Ti_3_C_2_ during the charging–discharging process [42]. In general, the charge storage mechanism of 3D V-, N-Ti_3_C_2_ includes the pseudocapacitive behavior of H^+^ and chemical absorption/desorption of Zn^2+^. Based on ex situ XRD and XPS, the charge storage mechanism of the 3D V-, N-Ti_3_C_2_ cathode probably behaves as follows:

Discharge:

C-Ti-O + H^+^ + e^−^ → C-Ti-OH; (2)

C-Ti-O + Zn^2+^ + 2e^−^ → C-Ti-O-Zn. (3)

Charge:

C-Ti-OH − e^−^ → C-Ti-O + H^+^; (4)

C-Ti-O-Zn − 2e^−^ → C-Ti-O + Zn^2+^. (5)

**Figure 5:**
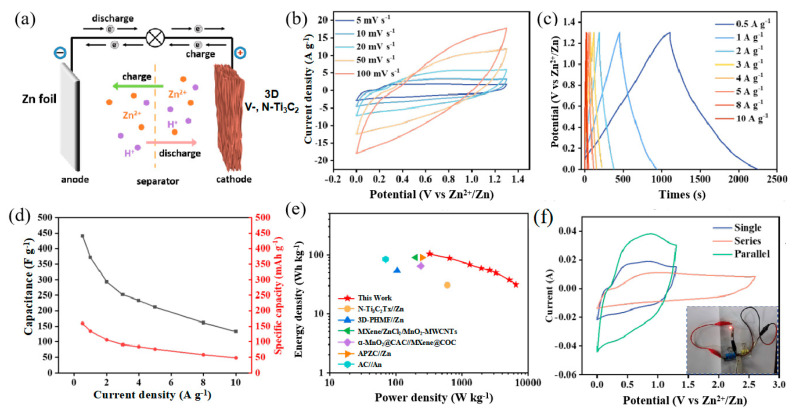
(**a**) Schematic diagram showing the discharging process of the 3D V-, N-Ti_3_C_2_//Zn ZIC. (**b**) CV curves of the 3D V-, N-Ti_3_C_2_//Zn ZIC from 5 to 100 mV s^−1^. (**c**) GCD curves of 3D V-, N-Ti_3_C_2_//Zn ZIC at different current densities. (**d**) Specific capacity and specific capacitance of the 3D V-, N-Ti_3_C_2_//Zn ZIC. (**e**) Ragone plots of the 3D V-, N-Ti_3_C_2_//Zn ZIC in comparison with other reported ZICs. (**f**) CV curves of the two ZICs in series and parallel.

To exemplify the viability of the 3D V-, N-Ti_3_C_2_ film for practical applications, ZIC devices were assembled with zinc sheet and 3D V-, N-Ti_3_C_2_ as anode and cathode, respectively (Figure 5a). The CV curves of the 3D V-, N-Ti_3_C_2_//Zn show a rectangular shape with weak redox peaks at 5 mV s^−1^ to 100 mV s^−1^ in Figure 5b, indicating the pseudo-capacitance feature of the 3D V-, N-Ti_3_C_2_//Zn ZIC. The GCD curves (Figure 5c) show symmetric charge/discharge processes at 0.5 to 10 A g^−1^, implying a high coulombic efficiency. The specific capacitance and specific capacity were able to reach up to 441 F g^−1^ and 159 mAh g^−1^, respectively, at 0.5 A g^−1^, implying an excellent energy storage performance (Figure 5d). Furthermore, we compared the energy density and power density of the 3D V-, N-Ti_3_C_2_//Zn with previously reported ZICs. As shown in Figure 5e, the 3D V-, N-Ti_3_C_2_//Zn ZIC 103 Wh kg^−1^ energy density was at a power density of 325 W kg^−1^, which was significantly superior to recently reported ZICs [29,34,45,46,51]. In addition, the 3D V-, N-Ti_3_C_2_//Zn ZIC full cells exhibited a favorable cyclic stability, which exhibited 86.6% capacity retention after 3000 cycles at 10 A g^−1^ (Figure S14). To further verify the viability of the 3D V-, N-Ti_3_C_2_//Zn ZIC, we extended the working voltage window and capacity by connecting two ZIC devices in parallel and in series. as depicted in Figure 5b. As anticipated, the voltage window widened to 2.6 V when two devices were connected in series, and then two devices in parallel were able to double the capacity. A small red LED bulb was able to be successfully powered by two 3D V-, N-Ti_3_C_2_//Zn ZICs connected in series, indicating that the ZICs can work effectively in practical applications.

## 4. Conclusions

In summary, the 3D V-, N-Ti_3_C_2_ film electrode with a high specific capacity was rationally designed by constructing 3D structures and using V/N dual-doping. Thanks to the porous structure design, heteroatom doping, and metal-ion pre-intercalation, the fabricated 3D V-, N-Ti_3_C_2_ based on Zn-ion pre-intercalation delivered a maximum capacitance of 855 F g^−1^ at 0.3 A g^−1^ and demonstrated 95.26% capacitance retention after 5000 charge/discharge cycles. The dual-ion energy storage mechanism of H^+^/Zn^2+^ was revealed by using ex situ XRD and XPS. In addition, the as-assembled aqueous 3D V-, N-Ti_3_C_2_//Zn ZICs displayed 103 Wh kg^−1^ energy density at a power density of 325 W kg^−1^ and a favorable cycling durability. This work presents an illuminating insight into rational pore structural design and heteroatom doping to obtain a desirable specific capacity and energy density for MXene electrode materials in energy storage systems.

## Data Availability

Data are contained within the article and Supplementary Materials.

## Supplementary Materials

The following supporting information can be downloaded at: https://www.mdpi.com/article/10.3390/nano14060490/s1, Figure S1. (a) SEM image few-layered Ti_3_C_2_T_x_ nanosheets, (b) XRD of Ti_3_AlC_2_ and Ti_3_C_2_T_x_. Figure S2. (a) SEM cross-sectional images, (b,c) the optical figures of Ti_3_C_2_ film. Figure S3. SEM cross-sectional images of 3D Ti_3_C_2_ film. Figure S4. SEM cross-sectional images of (a) V-Ti_3_C_2_, (b) V-, N-Ti_3_C_2_, and (c,d) 3D V-, N-Ti_3_C_2_ film. The inset is the optical figures of 3D V-, N-Ti_3_C_2_ film. Figure S5. N_2_ adsorption–desorption isotherms of 3D V-, N-Ti_3_C_2_ and V-, N-Ti_3_C_2_. Figure S6. (a) TEM images of the 3D V-, N-Ti_3_C_2_, (b) EDX elemental mapping of Ti, O and C for the 3D V-, N-Ti_3_C_2_. Figure S7. Survey XPS spectra of 3D V-, N-Ti_3_C_2_ film. Figure S8. High-resolution XPS spectra of (a) Ti 2p, (b) C 1s, (c) Zn 2p for the 3D V-, N-Ti_3_C_2_ film. Figure S9. (a) CV curves and (b) galvanostatic charge/discharge profiles of the Ti_3_C_2_ film. Figure S10. (a) CV curves and (b) galvanostatic charge/discharge profiles of the 3D Ti_3_C_2_ film. Figure S11. (a) CV curves and (b) galvanostatic charge/discharge profiles of the V-Ti_3_C_2_ film. Figure S12. (a) CV curves and (b) galvanostatic charge/discharge profiles of the V-, N-Ti_3_C_2_ film. Figure S13. Specific capacity and specific capacitance of the 3D V-, N-Ti_3_C_2_ film in 2M ZnSO_4_. Figure S14. Cycling performance of the 3D V-, N-Ti_3_C_2_//Zn ZIC at 10 A g^−1^. Figure S15. CV curves of the (a) Ti_3_C_2_ film, (b) 3D Ti_3_C_2_ film, (c) V-Ti_3_C_2_ film, (d) V-, N-Ti_3_C_2_ film, and (e) 3D V-, N-Ti_3_C_2_ film at different scanning rates, and (f) double-layer capacitance diagram of samples. Table S1. Comparison of storage energy performances between this work and previously reported in the literature. References [34,36,38,39,43,45,52] are cited in the Supplementary Materials.
