# Supplementary material for: Three-Dimensional Vanadium and Nitrogen Dual-Doped Ti3C2 Film with Ultra-High Specific Capacitance and High Volumetric Energy Density for Zinc-Ion Hybrid Capacitors
Source: Nanomaterials (Basel). 2024 Mar 8;14(6):490. doi: 10.3390/nano14060490 (PMC10974172; doi:10.3390/nano14060490)
Supplement: Supplementary file 1 [file nanomaterials-14-00490-s001.zip › nanomaterials-2878188-supplementary.pdf]

Supplementary Materials

Three Dimensional Vanadium and Nitrogen Dual-Doped Ti_3C_2 Film with Ultra-high Specific Capacitance and High Volume Energy Density For Zinc Ion Hybrid Capacitors

Xinhui Jin, Siliang Yue, Jiangcheng Zhang, Liang Qian, Xiaohui Guo*

Key Lab of Synthetic and Natural Functional Molecule Chemistry of Ministry of Education, The College of Chemistry and Materials Science, Northwest University, Xi'an 710069, P. R. China.

* Correspondence: guoxh2009@nwu.edu.cn

Characterization

The morphology of the samples was studied by field emission scanning electron microscopy (FE-SEM, Hitachi SU8010, Japan) and transmission electron microscopy (FEI Talos F200X, USA). The crystalline phases of the obtained products were characterized by X-ray diffraction on a Bruker D8 Advance (Bremen, Germany). The surface chemical compositions were analyzed by X-ray photoelectron spectroscopy (PHI5000 Versa Probe III, Japan). Nitrogen absorption/desorption isotherms of the samples were obtained at -196 °C using the Brunauer-Emmett-Teller (BET) method, and the samples were outgassed under vacuum for 6 h at 150 °C.

Electrochemical measurements

The electrochemical performance of all film electrodes was characterized by employing a two-electrode system with Zn foil as counter electrode and 2 M $ZnSO_4$ as electrolyte. The CHI 760E workstation was used to collect cyclic voltammogram (CV), electrochemical impedance spectroscopy (EIS), and galvanostatic charge/discharge curves (GCD). Equation (1) was used to calculate the specific capacitances (C_m) of active materials from the galvanostatic charge/discharge curves:

$$C_m = \frac{I \times \Delta t}{m \times \Delta V} \quad (1)$$

where I is the discharge current, m is the mass of active materials, Δt is the discharge time, and ΔV is the working voltage range.

The energy density (E) and power density (P) were calculated using the following equations:

$$E = \frac{1}{2 \times 3.6} C_m V^2 \quad (2)$$

$$P = \frac{E \times 3600}{\Delta t} \quad (3)$$

where C_m is the capacitance of film electrode, Δt is the discharge time, V is the operation potential window.

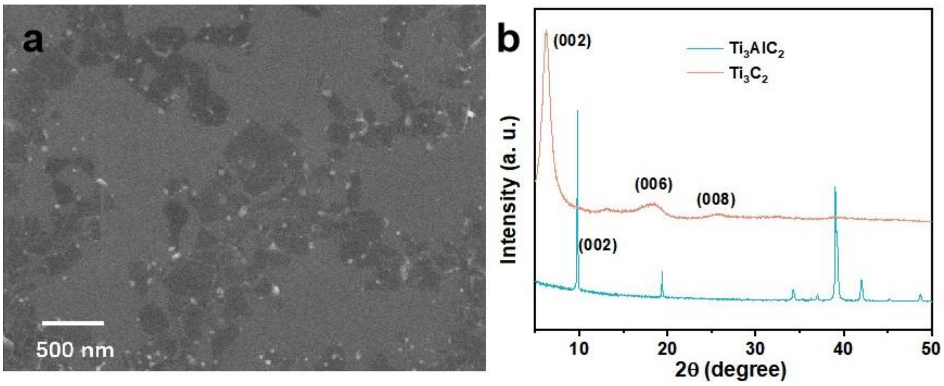

Figure S1. (a) SEM image few-layered $Ti_3C_2T_x$ nanosheets, (b) XRD of Ti_3AlC_2 and $Ti_3C_2T_x$.

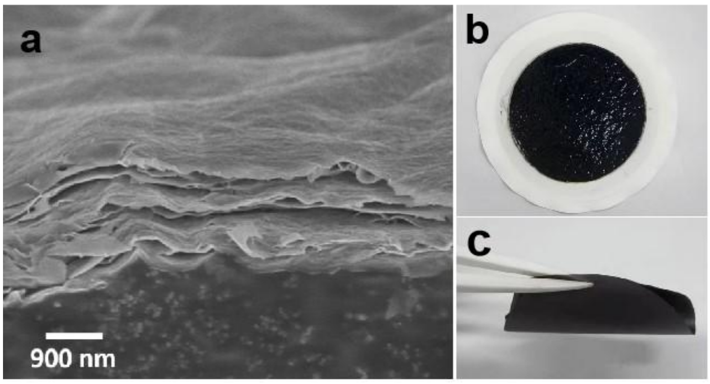

Figure S2. (a) SEM cross-sectional images, (b-c) the optical figures of Ti_3C_2 film.

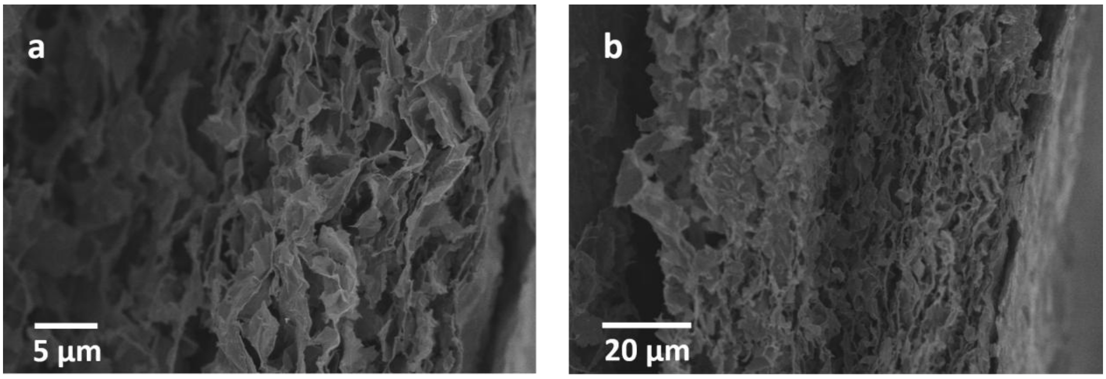

Figure S3. SEM cross-sectional images of 3D Ti_3C_2 film.

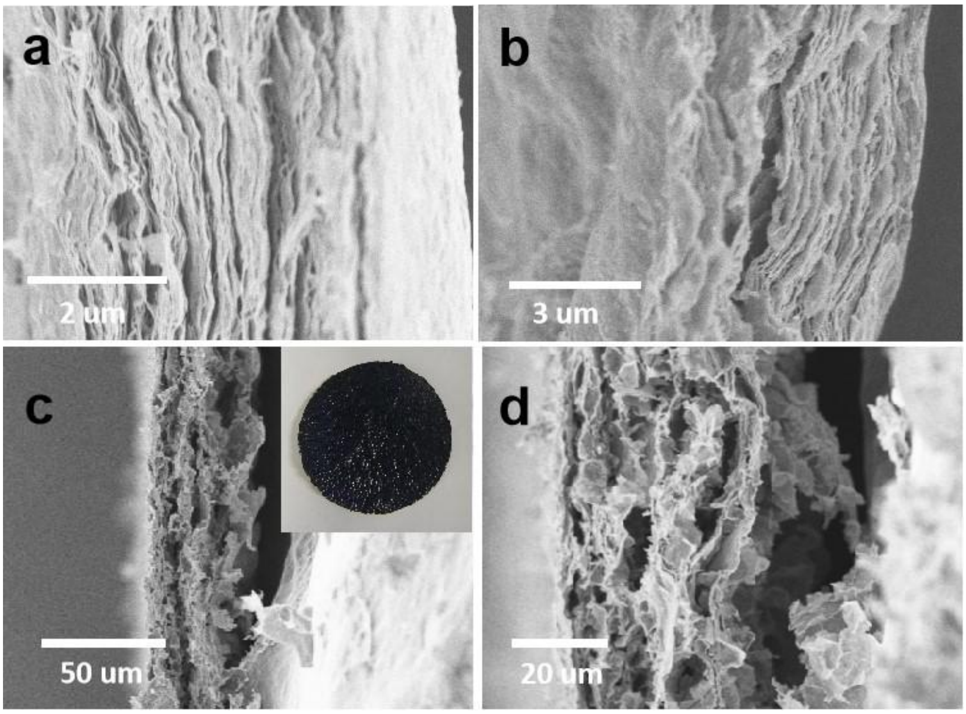

Figure S4. SEM cross-sectional images of (a) V-Ti_3C_2, (b) V-, N-Ti_3C_2, and (c-d) 3D V-, N-Ti_3C_2 film. The inset is the optical figures of 3D V-, N-Ti_3C_2 film.

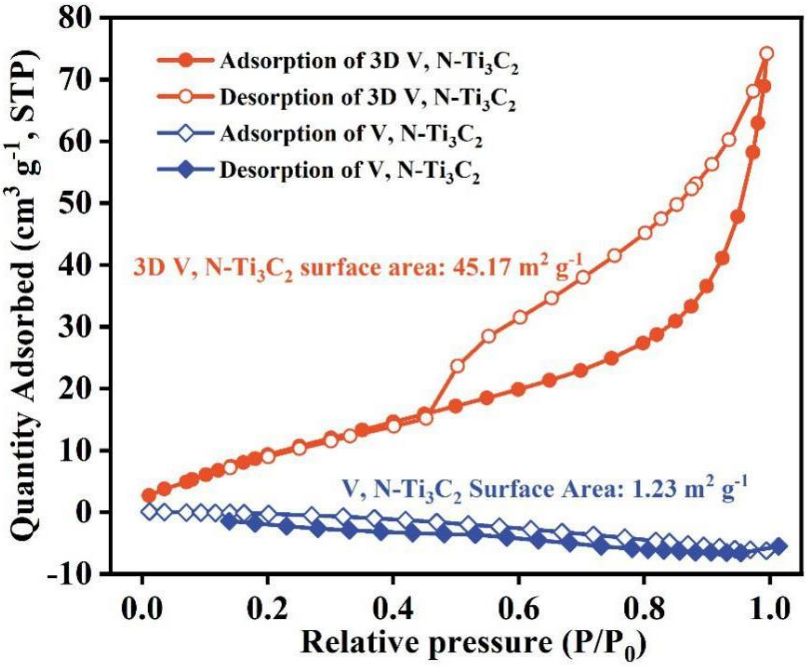

Figure S5. N_2 adsorption–desorption isotherms of 3D V-, N-Ti_3C_2 and V-, N-Ti_3C_2.

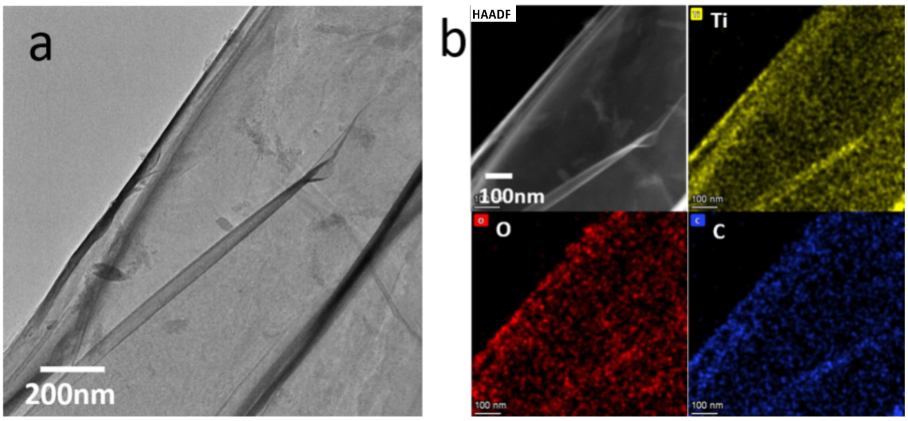

Figure S6. (a) TEM images of the 3D V-, N-Ti_3C_2, (b) EDX elemental mapping of Ti, O and C for the 3D V-, N-Ti_3C_2.

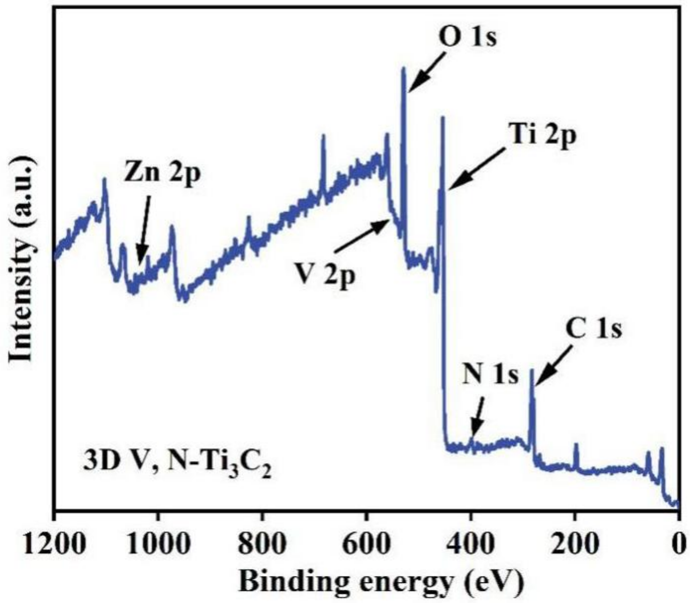

Figure S7. Survey XPS spectra of 3D V-, N-Ti_3C_2 film.

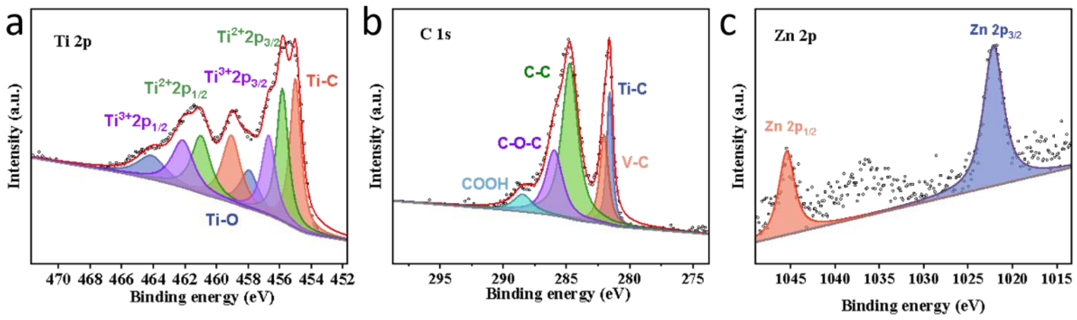

Figure S8. High-resolution XPS spectra of (a) Ti 2p, (b) C 1s, (c) Zn 2p for the 3D V-, N-Ti_3C_2 film.

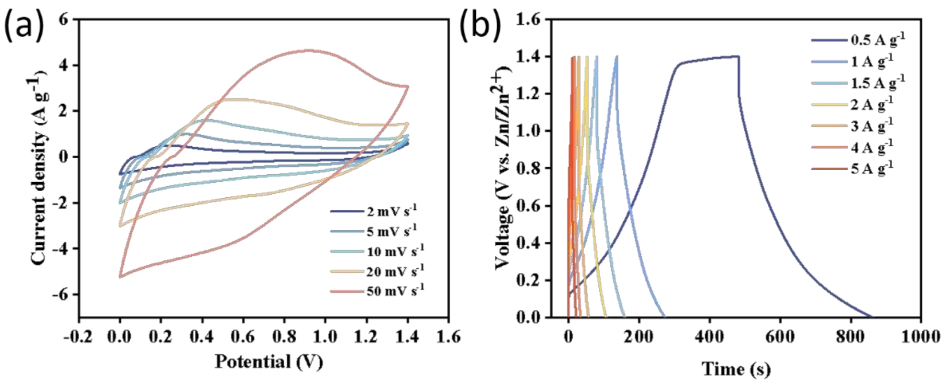

Figure S9. (a) CV curves and (b) galvanostatic charge/discharge profiles of the Ti_3C_2 film.

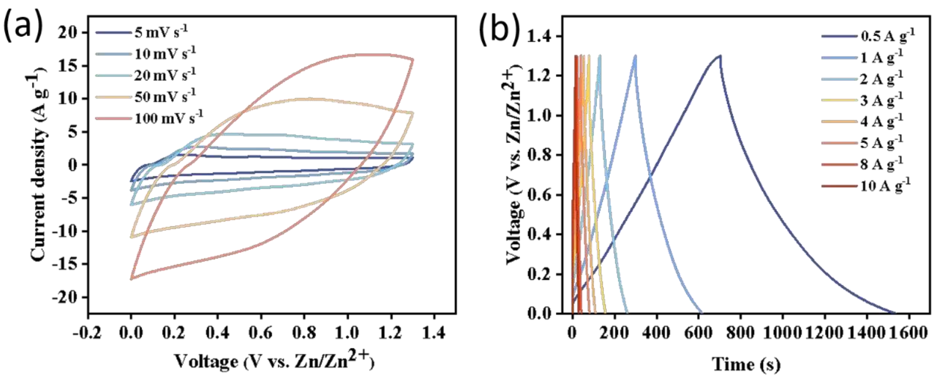

Figure S10. (a) CV curves and (b) galvanostatic charge/discharge profiles of the 3D Ti_3C_2 film.

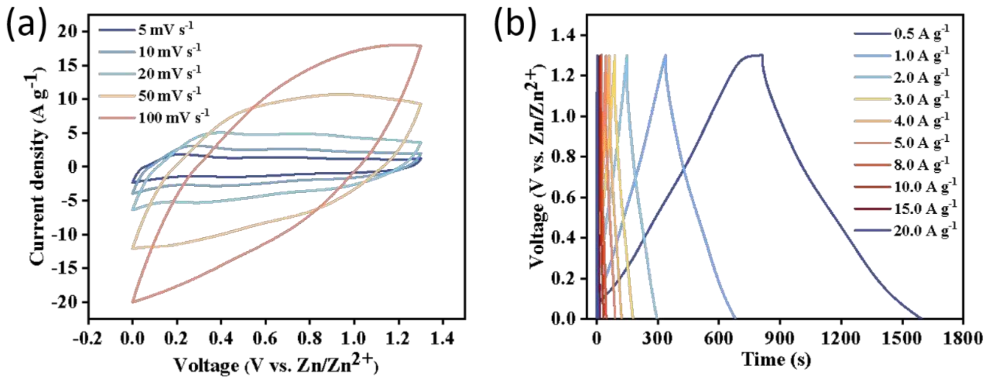

Figure S11. (a) CV curves and (b) galvanostatic charge/discharge profiles of the V-Ti_3C_2 film.

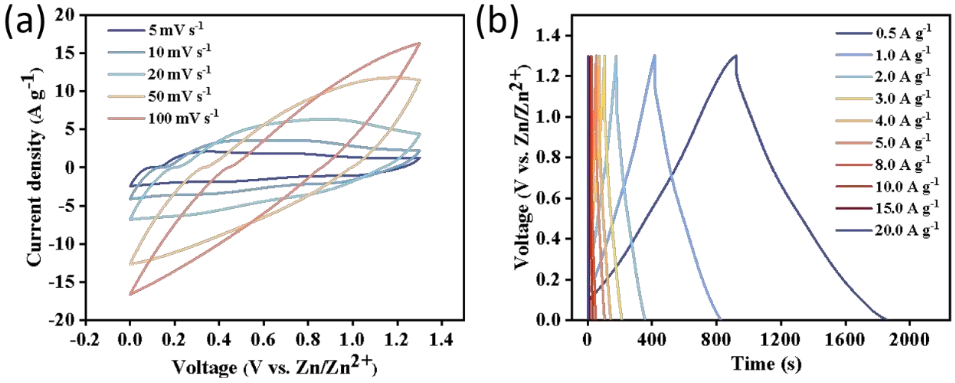

Figure S12. (a) CV curves and (b) galvanostatic charge/discharge profiles of the V-, N-Ti_3C_2 film.

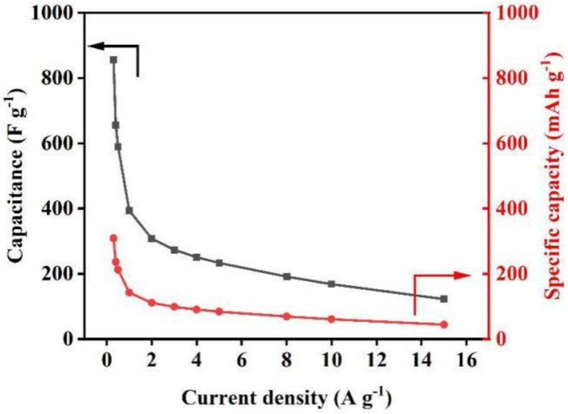

Figure S13. Specific capacity and specific capacitance of the 3D V-, N-Ti_3C_2 film in 2M $ZnSO_4$.

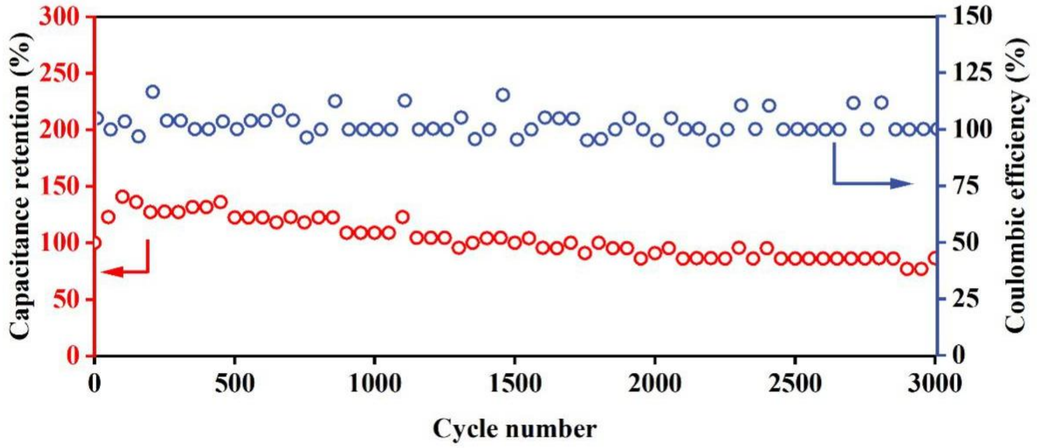

Figure S14. Cycling performance of the 3D V-, N-Ti_3C_2//Zn ZIC at 10 A g^{-1}.

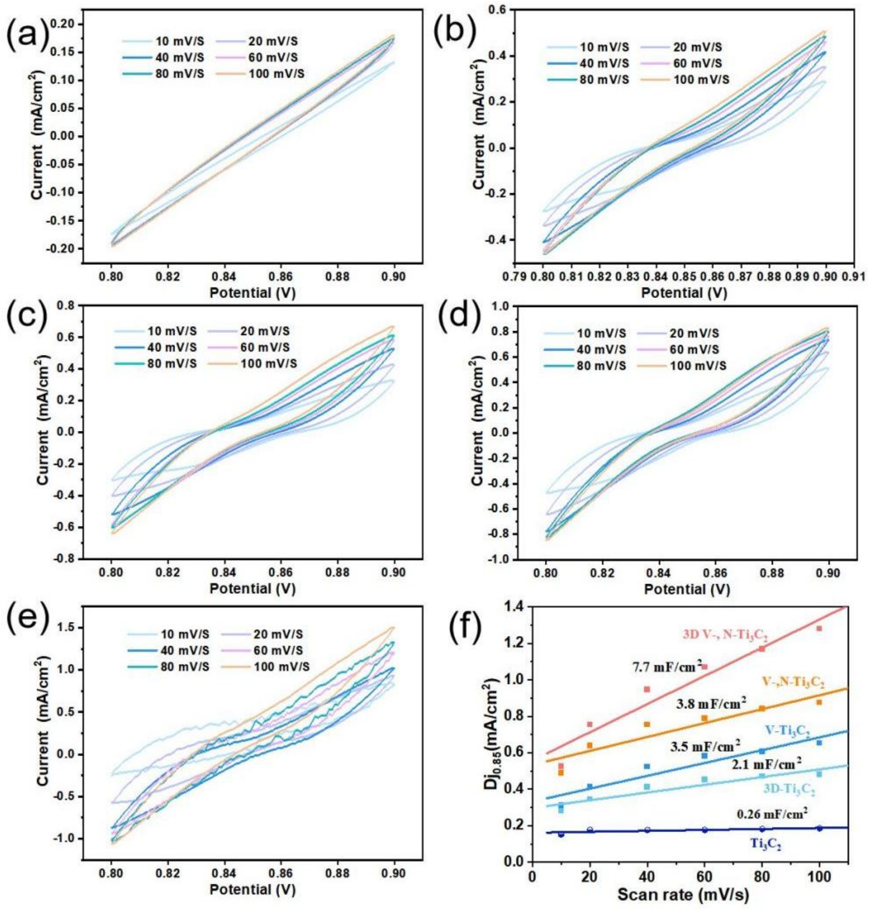

Figure S15. CV curves of the (a) Ti_3C_2 film, (b) 3D Ti_3C_2 film, (c) V-Ti_3C_2 film, (d) V-, N-Ti_3C_2 film, and (e) 3D V-, N-Ti_3C_2 film at different scanning rates, and (f) double-layer capacitance diagram of samples.

Table S1. Comparison of storage energy performances between this work and previously reported in the literature

Cathode	Anode	Electrolyte	Voltage （V）	Capacity	Current density （A/g）	Energy density (Wh Kg^{-1})	Ref.
AC	2D Zn	1M $ZnSO_4$	0.2-1.8	468 F/g	0.5	208	[S1]
AC	Zn	2M $ZnSO_4$	0-1.8	121 mAh/g	0.1	84	[S2]
HNPC	Zn	1M $ZnSO_4$	0-1.8	177 mAh/g	4.2	107	[S3]
N-HPC	Zn	2M $ZnSO_4$	0.2-1.8	136 mAh/g	0.1	191	[S4]
Ti_3C_2	Zn/Ti_3C_2	1M $ZnSO_4$/Gel	0-1.35	132 F/g	0.5	--	[S5]

3D porous H-$Ti_3C_2T_x$ films	Zn	2M $Zn(CF_3SO_4)_2$	0-1.3	105 mAh/g	0.2	53.6	[S6]
G-PANI	Zn	2M $ZnSO_4$	0.3-1.6	154 mAh/g	0.1	205	[S7]
**3D V-, N-Ti_3C_2**	**Zn**	**2M $ZnSO_4$**	**0-1.3**	**309 mAh/g 855 F/g**	**0.3**	**201**	**This work**

References:

[S1] An, G. H.; Hong, J.; Pak, S.; Cho, Y.; Lee, S.; Hou, B.; Cha, S. 2D Metal Zn Nanostructure Electrodes for High-Performance Zn Ion Supercapacitors. *Adv. Energy Mater.* **2019**, *10* (3), 1902981

[S2] Dong, L.; Ma, X.; Li, Y.; Zhao, L.; Liu, W.; Cheng, J.; Xu, C.; Li, B.; Yang, Q.-H.; Kang, F. Extremely safe, high-rate and ultralong-life zinc-ion hybrid supercapacitors. *Energy Storage Mater.* **2018**, *13*, 96-102.

[S3] Zhang, H.; Liu, Q.; Fang, Y.; Teng, C.; Liu, X.; Fang, P.; Tong, Y.; Lu, X. Boosting Zn-ion energy storage capability of hierarchically porous carbon by promoting chemical adsorption. *Adv. Mater.* **2019**, *31* (44), e1904948.

[S4] Liu, P.; Gao, Y.; Tan, Y.; Liu, W.; Huang, Y.; Yan, J.; Liu, K. Rational design of nitrogen doped hierarchical porous carbon for optimized zinc-ion hybrid supercapacitors. *Nano Res.* **2019**, *12* (11), 2835-2841.

[S5] Yang, Q.; Huang, Z.; Li, X.; Liu, Z.; Li, H.; Liang, G.; Wang, D.; Huang, Q.; Zhang, S.; Chen, S.; Zhi, C. A wholly degradable, rechargeable Zn–Ti_3C_2 MXene capacitor with superior anti-self-discharge function. *ACS Nano* **2019**, *13* (7), 8275-8283.

[S6] Li, F.; Liu, Y.-l.; Wang, G.-G.; Zhang, S.-Y.; Zhao, D.-Q.; Fang, K.; Zhang, H.-Y.; Yang, H. Y. 3D porous H-$Ti_3C_2T_x$ films as free-standing electrodes for zinc ion hybrid capacitors. *Chem. Eng. J.* **2022**, *435,* 135052.

[S7] Han, J.; Wang, K.; Liu, W.; Li, C.; Sun, X.; Zhang, X.; An, Y.; Yi, S.; Ma, Y. Rational design of nano-architecture composite hydrogel electrode towards high performance Zn-ion hybrid cell. *Nanoscale* **2018**, *10* (27), 13083-13091.
